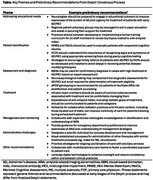# Delphi Consensus for Implementation of Anti‐amyloid mAb Initiation in Private Practice Neurology: Preliminary Recommendations From Experienced Providers

**DOI:** 10.1002/alz70861_108789

**Published:** 2025-12-23

**Authors:** David C Weisman, Bela Ajtai, Sarah Harlock, Michael Hemphill, Cara Leahy, Justin S Moon, Salvatore Q Napoli, Jose Soria‐Lopez, Jeffrey Gelblum

**Affiliations:** ^1^ Abington Neurologic Associates, Abington, PA USA; ^2^ Dent Neurologic Institute, Amherst, NY USA; ^3^ Savannah Neurological Specialists, Savannah, GA USA; ^4^ Memorial Healthcare Institute for Neuroscience, Owosso, MI USA; ^5^ Denver Neurological Clinic, Lone Tree, CO USA; ^6^ Neurology and Infusion Centers of New England, Foxborough, MA USA; ^7^ The Neuron Clinic, San Diego, CA USA; ^8^ First Choice Neurology, Medley, FL USA

## Abstract

Approval of anti‐amyloid monoclonal antibodies (mAbs) has altered the Alzheimer’s disease (AD) therapeutic landscape, but implementation of these therapies within private practice neurology has been hindered by a lack of established guidelines. The Delphi technique is a well‐established methodology for developing consensus recommendations and has been previously used within the AD field. Here, we report preliminary recommendations from a Delphi consensus process focused on implementation of anti‐amyloid mAbs in private practice neurology. The Delphi consensus panel includes 9 expert clinicians and administrators with experience implementing anti‐amyloid mAbs within private practice neurology; the panel is overseen by 2 Delphi chairs. Initial recommendations were developed based on insights from an initial survey and panel discussion and subsequently vetted for clarity by the panelists as a first step in the Delphi process. These recommendations generally focused on 6 main areas in which panelists faced the greatest challenges (Table). Recommendations to address educational needs included those related to education for patients/caregivers, primary care physicians, neurology medical staff, administrators, and insurers/payers. Barriers to patient identification may be lessened with standardization of screening tools and expanded training of medical staff (eg, nurses, medical assistants, advanced practice providers). Recommendations for assessment and diagnosis of patients with AD emphasized the importance of comprehensive assessment and use of all accessible resources (eg, amyloid positron emission tomography, cerebrospinal fluid testing, blood‐based biomarkers). Panelists noted that recommendations for treatment administration differ among available anti‐amyloid mAbs; refer to the US Food and Drug Administration–approved prescribing information. Management and monitoring should be conducted according to prescribing information and appropriate use guidelines in collaboration with other specialists. Recommendations to address administrative challenges highlighted the importance of implementing specific roles for care coordinators and process managers, as well as creating checklists to reinforce standardized procedures. Additional recommendations highlighted the importance of applying novel solutions to expand treatment outreach, including to underserved populations. The Delphi consensus recommendations are designed to address barriers faced by private practice neurologists initiating treatment with anti‐amyloid mAbs, and are intended to improve access to mAb therapies for appropriate patients with AD.